# Multisite Lifestyle for Older People after the Fukushima Nuclear Disaster

**DOI:** 10.3390/geriatrics8050087

**Published:** 2023-09-03

**Authors:** Naomi Ito, Isamu Amir, Hiroaki Saito, Nobuaki Moriyama, Ayako Furuyama, Priya Singh, Stephanie Montesino, Chika Yamamoto, Mika Sato, Toshiki Abe, Tianchen Zhao, Masaharu Tsubokura

**Affiliations:** 1 Department of Radiation Health Management, Fukushima Medical University School of Medicine, 1 Hikariga-oka, Fukushima 960-1295, Japan; iamir@fmu.ac.jp (I.A.); hiros@fmu.ac.jp (H.S.); cy911212@fmu.ac.jp (C.Y.); a-toshi@fmu.ac.jp (T.A.); cho1230@fmu.ac.jp (T.Z.); tsubo-m@fmu.ac.jp (M.T.); 2 Department of Internal Medicine, Soma Central Hospital, Okinouchi, Soma 976-0016, Japan; 3 Department of Public Health, Fukushima Medical University School of Medicine, 1 Hikariga-oka, Fukushima 960-1295, Japan; moriyama@fmu.ac.jp; 4 Health Promotion Center, Fukushima Medical University, 1 Hikariga-oka, Fukushima 960-1295, Japan; a-furuya@fmu.ac.jp; 5 Icahn School of Medicine at Mount Sinai, 1 Gustave L. Levy Place, New York, NY 10029, USA; priya.singh@icahn.mssm.edu (P.S.); stephanie.montesino@icahn.mssm.edu (S.M.); 6 Department of Health Nursing of International Radiation Exposure, Fukushima Medical University School of Medicine, 1 Hikariga-oka, Fukushima 960-1295, Japan; samika@fmu.ac.jp

**Keywords:** disaster, Fukushima, nuclear incident, return policy, multisite living, ageing society, ageing in place, public reconstruction housing, older people

## Abstract

After the Fukushima nuclear power plant disaster in 2011, the Japanese government implemented a return policy, lifting most evacuation orders in former evacuation areas. Consequently, the return of residents is currently underway. However, it has become common for a large number of residents to carry out multisite living, a lifestyle involving returning to their hometown while maintaining their house at the evacuation site, or living at more than two sites. This report focuses on one aspect of the secondary effects of the nuclear incident, which forced affected residents to adopt a multisite lifestyle. Disasters always have a strong impact, via displacement, on those who are socially vulnerable, such as older people in an ageing society. They need intense support to resume their daily life as it was before the incident. For this report, we interviewed an elderly lady in her 90s, who is executing “multisite living” at evacuation sites, in order to obtain reassurance from neighbours and the local community. Our findings may provide valuable suggestions on how older people can restart their lives with the local community in an ageing society after disasters, which could apply to any kind of disaster preparedness.

## 1. Introduction

The desire to live in a familiar place is a universal aspiration [1,2]. This is also the central concept of living in a community, and the idea of “ageing in place” [3,4]. Residing in a familiar community helps maintain and enhance a person’s independence, dignity, and quality of life [5]. However, this ability is influenced by several factors, such as disasters and disease. Despite the significance of this aspiration, adequate measures to address it are not often implemented during emergencies. Determining whether appropriate measures can be implemented under such circumstances, for the realisation of ageing in place, is an important public health issue.

Achieving ageing in a place during a disaster can be a challenging situation, due to various factors [6,7]. One such factor is the tendency of younger generations to establish new lives in evacuation destinations, whereas older generations prefer to remain in places where they have lived for many years [8,9]. This creates concerns about the emotional distress experienced by residents when they must relocate and adapt to new environments, leading to lifestyle changes [10]. Furthermore, post-disaster areas often face rapid population decline and ageing [11,12], which has garnered national and international attention regarding the preservation of residents’ quality of life [13]. Although countermeasures are crucial in disaster situations, the implementation of strategies to support ageing in place remains insufficient.

On 11 March 2011, a catastrophic event occurred, when the Great East Japan Earthquake struck with a magnitude of 9.0. Within an hour, a devastating tsunami occurred. Owing to these events, Units 1, 3, and 4 of the Fukushima Daiichi Nuclear Power Plant (FDNPP), operated by the Tokyo Electric Power Company, lost power. The loss of power rendered the reactors unable to cool, leading to explosions caused by the hydrogen generated at high temperatures [14]. Consequently, radioactive material was released and dispersed outside the power plant, in the northwest direction. In response, the Japanese government declared a nuclear emergency, and issued an evacuation order for residents within 30 km of the reactor. Residents were promptly evacuated following the order. Subsequently, the evacuation order was gradually and partially lifted over time. However, many residents were unable to return to their original homes [15]. Some of them decided to reside in the places where they had sought refuge during the evacuation.

**Figure 1:**
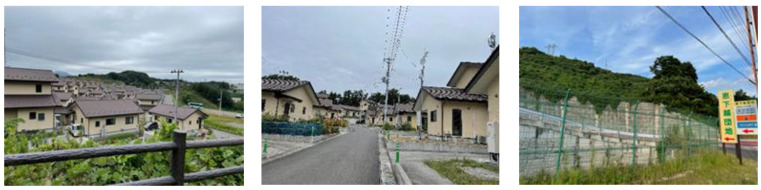
Left: the reconstruction public housing for Katsurao villagers, which was built on a hilltop in Miharu Town; centre: a road located within the housing area; right: an entrance toward the reconstruction public housing, with the signboard on the right indicating “Egenokoshi Danchi”.

Katsurao Village, located approximately 20–30 km from the FDNPP, is a mountainous village with a population of approximately 1400. When the disaster struck, most residents evacuated to Miharu Town and Koriyama City. In the same year, temporary housing complexes were constructed in Miharu Town (Figure 1). People living away from their home village began to show various secondary health effects [16,17,18,19]. Meanwhile, the evacuation order for Katsurao Village was lifted on a large scale in June 2016, and the residents have gradually been returning home. Presently, the number of returnees in the village is 324 (an ageing rate of 57%), representing 25.2% of the village’s registered population (1298 as of 1 May 2023). As many residents continue to stay at the evacuation site [20,21], an understanding of the situation of these evacuated residents is crucial to Japan’s return policy [22,23]. Since the lifting of the evacuation order in 2016, the village has implemented various measures to assist residents in rebuilding their lives [24]. Nevertheless, many individuals maintain a multisite lifestyle, which includes their original house in the village, and a new house at an evacuation site.

The multisite lifestyle (living) in this report refers to a lifestyle involving living at multiple sites following a nuclear incident. Few studies have described situations in which large numbers of residents feel compelled to maintain their residences in multiple locations, amidst the ongoing return policies [25]. Why do so many people maintain multiple sites of residence after a nuclear accident? The aim of this report is to provide insights into the present situation, and challenges associated with the multisite lifestyle experienced by residents after a nuclear incident. This is to gain a better understanding of the current conditions and difficulties they encounter and, subsequently, utilise these findings to support promotion of the health of residents experiencing evacuation.

## 2. Methodology and Data Collection

This case report was based on interviews, following observations and the selection of residents, to capture real-life social phenomena, and enhance the internal validity of the study [26].

To clarify our research questions, we believe that conducting interviews is the best approach to bring forth the real voice of the residents. Although a questionnaire can also be useful, it cannot bring out the actual thoughts of the residents. Therefore, we carried out interviews with individual residents who were executing multisite living. The interviewees were selected via the following procedure.

The first author was engaged in cooperation with Katsurao Village as a public health nurse, to undertake health check-ups and home visits to acquire the health information of residents. Indeed, the first author participated in regional healthcare meetings once a month, to share health information, and discuss how to provide public health support. The first author studied the living situation of individual residents through observations, including daily conversations related to health maintenance at health check-up sites and home visits, and information shared at the meeting. As a result, the first author noticed and recognised the multisite living practised by Katsurao residents. Based on the research questions, the first author started interviewing residents who continued multisite living (20 of a total 200 residents). Among the 20 individuals whom we interviewed, we identified a woman representing a typical case of an older person living in multiple locations after the nuclear incident.

The interview was held face-to-face at the resident’s home, over three sessions in two months. The interview questions were as follows:

(i) How did you evacuate after the nuclear accident and up to the present?
(ii) How has your life changed with the evacuation and relocation?
(iii) The evacuation order has been lifted, but why don’t you return to Katsurao Village?
(iv) What are the good things and problems of living in multiple locations?
(v) Do you have any health concerns?
(vi) Are you satisfied with your current lifestyle?

We analysed the interview responses, and the results are summarised in the case presentation section.

Egenokoshi Housing Complex in Miharu Town (Figure 1) is a public reconstruction housing for residents of Katsurao Village who were evacuated after the nuclear incident. The move-in began in April 2016. Seventy houses in the complex are occupied by 135 residents, and thirty-six houses are vacant. Among the 135 residents, 57 are aged 65 years and older (ageing rate: 42.2%). There are 22 elderly people living alone (22 houses), and 10 houses where only older people live (data as of 1 June 2022).

A staff member of the Katsurao Village office is stationed in a unit of the complex, and visits all the houses every day.

## 3. Case Presentation

**Figure 2:**
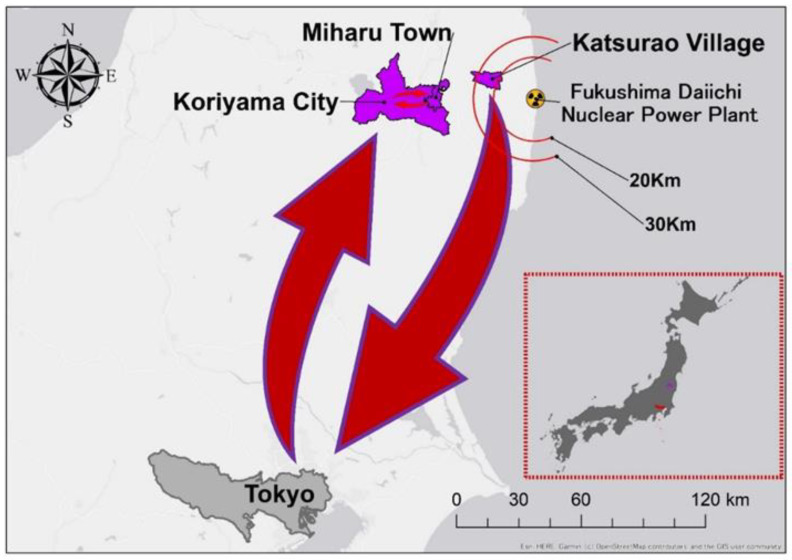
An elderly lady from Katsurao Village evacuated to Tokyo after the incident occurred in 2011, where she lived for approximately 8 years. She then purchased a house in Koriyama City. After living there for 2 years, her husband died, and she became alone. While she has kept her house in Koriyama, her children let her move to the public reconstruction housing complex in Miharu Town, due to their worries about their mother’s ability to live alone in Koriyama.

A woman in her 90s lived in Katsurao Village with her husband before the incident in 2011. After the nuclear plant incident, her son, who resided in Tokyo, rushed to pick them up immediately. They evacuated from their village to a distant location, they then resided as evacuees in municipal housing in Tokyo for the following 8 years (Figure 2). Because all three of their children lived in Tokyo, it was convenient for the couple to live there and receive support from their children. They made up their mind not to return to their village, because she and her husband were afraid of radiation exposure, derived from radioactive materials which scattered from the Fukushima nuclear power plant and covered their village. Their participation in anti-nuclear action provided the inspiration for the notion stated above.

After 8 years had passed in Tokyo since their evacuation, they purchased a new house in Koriyama City, and moved there (Figure 2). Their reason for the purchase was to live close to the graves of their ancestors, which are located in their village where her former home had already been deconstructed. Two years after the movement, her husband died from illness, and she became alone. Although she was able to live physically independently, she felt alone because there were no communities she belonged to at the new location, as they were different from those in her original village. Concerned about her wellbeing, her children let her move to a residence in a public reconstruction housing for Katsurao Village residents located in Miharu Town—the Egenokoshi Housing Complex (Figure 2). Fortunately, there was a vacant unit next to her sister’s residence in the complex, allowing her to move immediately. Consequently, she was able to build a relationship with the neighbours, including her sister. These relationships form the base of the local community network.

Currently, she lives in two locations, mainly in Miharu Town, while maintaining her home in Koriyama City. The reconstruction public housing where she moved was built to provide housing for Katsurao residents after the evacuation order was lifted. Her daughter who lived in Tokyo came to Fukushima prefecture to take care of her. During her 10-day stay, they travelled back and forth between Koriyama and Miharu. After her daughter left, the elderly woman continued living in Miharu. On her life there, she says, “I feel at ease here because there are people from the village”; “I feel reassured that people check on me every day”; and “Rental fee is cheap, less than JPY 10,000 (app. USD 70), good for a pensioner”. Recently, she was certified as a person requiring long-term care, and she attends daycare once a week. She talks about her life in Miharu Town as follows: “It is good for me that the daycare service car picks me up”; “I can still do things around me”; and “I want to live here for the rest of my life”.

Judging from her comments about her life in Miharu Town, she appears satisfied with her living circumstances and lifestyle; in particular, she feels comfortable with her relationship with her neighbours. Indeed, she accepts moving between two locations. As she wants to live in Miharu Town, she will not return to her original village.

## 4. Discussion

Text analysis was performed on the interview that we conducted. Text analysis includes not only the text itself, but also the context related to the situations the participant had faced, and her background.

This case is typical of nuclear disasters, where multiple sites continue to be maintained, including evacuation centres and places of origin. Nuclear disasters differ significantly from others, such as tsunamis and typhoons; they involve concerns about radioactive contamination, which leads evacuees to leave their original residence for a long period [27,28,29,30]. The evacuation order described here lasted longer than the evacuees’ expectation. Therefore, evacuees searched for houses at the evacuation sites, as compensation for nuclear disaster evacuees and housing subsidies were paid [31]. Indeed, as they thought that they would never be able to go back to their original land, many residents continued to live at their evacuation site even after the evacuation order had been lifted [32]. In another aspect, the special law for nuclear disaster evacuees helped to establish life at evacuation sites [33]. However, many of the evacuees still miss their original land, which has the same landscape as before. Therefore, they commute back to their original homes for work, maintain them, and restart their customs at their original site. That is why evacuees still carry out multisite living, 12 years after the nuclear incident occurred.

In addition to the description above, the following multiple challenges faced by an elderly woman resulted in multisite living: firstly, she was forced to relocate during her old age due to the nuclear incident. Secondly, her husband passed away, which led to her living alone. Leaving a familiar place of residence in old age, and the death of a companion, often lead to social isolation [34]. In other words, it is difficult for elderly people to build a community relationship after relocation. Indeed, it is possible that older people may migrate to seek social resources, such as medical, nursing, and preventive care, in line with the health problems they face. As a result of seeking a place to improve their quality of life, they opt to maintain multiple bases, such as the original place, the evacuation site, and the nursing home [25].

Over the course of repeated displacement, the follow-up and social support for residents’ health problems can be lacking [35,36]. In this case, however, public housing for disaster victims has made it possible for the elderly to build new relationships with familiar people, and to live close to the affected areas [37,38,39]. Intangible support, such as life support staff calls, informal support among residents, and shuttle buses to and from hospitals have been very helpful. Therefore, it is possible to bring about a happy and independent life, even if it is not in one’s original home [40].

This study has some limitations. The case report described here is only one of the 20 cases in Katsurao Village that we have encountered, and it is limited to the older population. To address these limitations, we believe that the study should be expanded to include surrounding areas and different age groups.

## 5. Conclusions

Our findings, through an interview, are the results of the forced relocation of victims after the nuclear incident, subsequent recovery, and local measures under the national return policy. The immediate challenges, including the health problems seen among the survivors, led residents to live in multiple locations, and the post-disaster social security policy made this possible. Reconstruction housing complexes led older people to settle, and allowed them to develop relationships with neighbours from the same village, which helped them to restart their lives in a familiar community after the disaster. This is applicable not only to disaster preparedness, but also in normal settings with rapid ageing.

Regarding the difficulties encountered, this region is advanced because of the population’s rapid aging and decline. We think that the findings of our research can provide a number of recommendations for global concerns with a similar regional component.

## Data Availability

Not applicable.
